# Supplementary material for: Androgen receptor contributes to radioresistance through DNA repair and autophagy in AR-positive prostate cancer cells
Source: bioRxiv. 2025 Dec 5:2025.12.03.690226. Preprint. [Version 1] doi: 10.64898/2025.12.03.690226 (PMC12822795; doi:10.64898/2025.12.03.690226)

bioRxiv preprint doi: https://doi.org/10.64898/2025.12.03.690226; this version posted December 5, 2025. The copyright holder for this preprint (which was not certified by peer review) is the author/funder, who has granted bioRxiv a license to display the preprint in perpetuity. It is made available under a CC-BY 4.0 International license.

Androgen Receptor Contributes to Radioresistance through DNA repair and autophagy in AR-Positive Prostate Cancer Cells

Ken-ichi Kudo, Qianhua Feng, Maria Isabel Chosco *et al.*

SUPPLEMENTARY INFORMATION

bioRxiv preprint doi: https://doi.org/10.64898/2025.12.03.690226; this version posted December 5, 2025. The copyright holder for this preprint (which was not certified by peer review) is the author/funder, who has granted bioRxiv a license to display the preprint in perpetuity. It is made available under a CC-BY 4.0 International license.

Supplementary Figure legends

Supplementary table 1. Primary and secondary antibody list
Supplementary table 2. Products used in siRNA knockdown experiment
Supplementary table 3. Sequence of primers used in RT-qPCR

Supplementary Figure 1. A, The plots of flow cytometry from selection of cells to detection of γH2AX signals. **B,** Comparison of γH2AX frequencies generated in HEK293T between high glucose (HG) and low glucose (LG) during 24 hours after irradiation. **C,** Representative profiles for cleaved caspase-3 (CC3) frequencies in HEK293T, LNCaP, and PC3. The numbers listed in each panel mean the median value. **D,** Side scatter (SSC) ratio calculated from SSC-A values in irradiated and unirradiated groups. *, $P<0.05$, **, $P<0.01$ vs. 0Gy.

Supplementary Figure 2. A, mRNA expression levels of *CDKN1A*, *GADD45A*, and *TP53* genes in LNCaP cells. Enz and Abi mean enzalutamide and abiraterone acetate. Data represent the mean values of two RT-qPCR assays. **B** and **C,** ICC images of LNCaP cells stained with (**B**) fluorescent azide targeting EdU incorporated in DNA strands and (**C**) anti-Ki67 antibody. **D,** mRNA expression levels of Exon 4 and cryptic exons (*CE3* and *CE5*) in AR gene. No. 1-6 in graph show the lot number of LNCaP cells. **E,** Relative mRNA expression levels of *ATG2A* gene in LNCaP cells. Data represent the means and SEs of three independent experiments. *, $P<0.05$, **, $P<0.01$.

Supplementary Figure 3. A, MDC profiles got with FCM. **B,** mRNA expression levels of p53-downstream genes in HEK293T, LNCaP, and PC3. The cells were treated in the same manner as MDC assay. The data show the fold change of each mRNA expression against negative control (CQ−, Rapa−, IR−). **C,** Heatmap showing differentially expressed autophagy-related gene expression in unirradiated (0 Gy) and irradiated (8 Gy). LNCaP cells were cultured in RPMI1640 containing 25 mM glucose (HG) or 5 mM glucose (LG). **D,** Phosphorylated AMPKα (Thr172; p-AMPKα) and dual phosphorylated p38 (Thr180/Tyr182; p-p38) were measured with western blot analysis, which are representative markers of mitogen-activated protein kinases (MAPK). **E,** p-AMPKα and AMPKα measured in PC3, LNCaP and HEK293T. GAPDH or βActin was used as an internal control.

Supplementary Figure 4. IHC images of patient-derived explants. (**A**) H&E staining images, (**B**) AR, (**C**) PSA, and (**D**) CK8 stained by DAB. (**E**) IHC-F of CK8 and γH2AX after irradiation. (**F**) Representative image showing QuPath-based cell segmentation in PCa tissue. Nuclei were stained with DAPI (blue), and cell segmentation was performed using the DAPI channel as a reference in QuPath. Detected cell boundaries are outlined in red.

bioRxiv preprint doi: https://doi.org/10.64898/2025.12.03.690226; this version posted December 5, 2025. The copyright holder for this preprint (which was not certified by peer review) is the author/funder, who has granted bioRxiv a license to display the preprint in perpetuity. It is made available under a CC-BY 4.0 International license.

Supplementary Table 1

Antigen	Clone	Supplier	Conjugation	Catalog no.	Specie	Experiment	Conc.
Primary antibodies							
LC3B	polyclonal	Thermo Fisher	unconjugate	PA1-46286	Rb	WB, IHC-F	1:1000,
CK8	EP1628Y	Abcam	unconjugate	53280	Rb	IHC	1:200
ATG9A	EPR2450(	Abcam	unconjugate	108338	Rb	IHC-F	1:50
AR	AR441	Abcam	unconjugate	9474	Ms	IHC-F	1:50
CD326	VU-1D9	Bio-Rad	unconjugate	MCA1870G	Ms	ICC	1:200
Phospho-AMPKα	-	Cell Signaling	unconjugate	2531	Rb	WB	1:1000
GAPDH	14C10	Cell Signaling	unconjugate	2118	Rb	WB	1:1000
Isotype control for	DA1E	Cell Signaling	AF488	2975	Rb	FCM	1:100
Cyclin D1	92G2	Cell Signaling	unconjugate	2978	Rb	WB	1:1000
Isotype control for CC3	DA1E	Cell Signaling	AF647	2985	Rb	FCM	1:100
Phospho-Akt (Ser473)	D9E	Cell Signaling	unconjugate	4060	Rb	WB	1:2000
Phospho-p38	D3F9	Cell Signaling	unconjugate	4511	Rb	WB	1:1000
CK8/18	C51	Cell Signaling	unconjugate	4546	Ms	IHC	1:50
βActin	13E5	Cell Signaling	unconjugate	4970	Rb	WB	1:1000
AR	D6F11	Cell Signaling	unconjugate	5153	Rb	IHC	1:800
PSA	D6B1	Cell Signaling	unconjugate	5365	Rb	IHC	1:400
Phospho-Rb	D20B12	Cell Signaling	unconjugate	8516	Rb	WB	1:1000
Rad51	D4B10	Cell Signaling	unconjugate	8875	Rb	WB	1:1000
Cleaved Caspase-3	D3E9	Cell Signaling	AF647	9602	Rb	FCM	1:100
Cleaved Caspase-3	D3E9	Cell Signaling	unconjugate	9661	Rb	IHC-F, ICC	1:200, 1:800
Phospho-H2A.X	20E3	Cell Signaling	unconjugate	9718	Rb	IHC-F, ICC	1:200
Phospho-H2A.X	20E3	Cell Signaling	AF488	9719	Rb	FCM	1:100
AR-V7	E3O8L	Cell Signaling	unconjugate	19672	Rb	WB	1:1000
DNA-PKcs	E6U3A	Cell Signaling	unconjugate	38168	Rb	WB	1:1000
Phospho-DNA-PKcs	E9J4G	Cell Signaling	unconjugate	68716	Rb	WB	1:1000
Secondary antibodies							
IgG		Cell Signaling	unconjugate	7076	Ms	WB	1:3000-5000
IgG		Cell Signaling	unconjugate	7074	Rb	WB	1:3000-5000
IgG		abcam	AF555	150114	Ms	IHC-F/ICC	1:200
IgG		ThermoFisher	AF488	A11008	Rb	IHC-F/ICC	1:500

Western blot: WB, Flow Cytometry : FCM, Fluorescent immunohistochemistry: IHC-F, immunocytochemistry: ICC, Ms: Mouse, Rb: Rabbit,

bioRxiv preprint doi: https://doi.org/10.64898/2025.12.03.690226; this version posted December 5, 2025. The copyright holder for this preprint (which was not certified by peer review) is the author/funder, who has granted bioRxiv a license to display the preprint in perpetuity. It is made available under a CC-BY 4.0 International license.

Supplementary Table 2

siRNA targeting gene	Target exon	Product company	Cat#
AR	Exon 2	ThermoFisher Scientific	4390824, s1538
AR	Exon 7		4390824, s1539
ATG2A	Exon 32		4392420, s23097
BECN1	Exon 5-7		4392420, s16537
MAP1LC3B	Exon 2 and 3		4392420, s37749

bioRxiv preprint doi: https://doi.org/10.64898/2025.12.03.690226; this version posted December 5, 2025. The copyright holder for this preprint (which was not certified by peer review) is the author/funder, who has granted bioRxiv a license to display the preprint in perpetuity. It is made available under a CC-BY 4.0 International license.

Supplementary Table 3

Gene	Forward 5'->3'	Reverse 5'->3'	Applications
AR	CTC TCA CAT GTG GAA GCT GC	AGC CTC TCC TTC CTC CTG TA	Prostate marker
KLK3/PSA	CGG ATG CTG TGA AGG TCA TG	CTT CTG AGG GTG AAC TTG CG	
BAX	ACC AAG AAG CTG AGC GAG TG	CAG TTG AAG TTG CCG TCA GA	DNA damage response marker
CDKN1A	CAG CAG AGG AAG ACC ATG TG	GGC GTT TGG AGT GGT AGA AA	
GADD45A	ACG AGG ACG ACG ACA GAG AT	GCA GGA TCC TTC CAT TGA GA	
ATG2A	CCA GTT CCG GTC AGA GCT TA	CAG TTC CTC TCC CTT CTC CG	Autophagy marker
BECN1	GAC ACT CAG CTC AAC GTC AC	GCT CCA TCT GTA ACT GTT CAC T	
MAP1LC3B	AAG ACC TTC AAG CAG CGC	TCA CCA ACA GGA AGA AGG CC	
β-Actin	GAA GTG TGA CGT GGA CAT CC	CCG ATC CAC ACG GAG TAC TT	Internal standard

bioRxiv preprint doi: https://doi.org/10.64898/2025.12.03.690226; this version posted December 5, 2025. The copyright holder for this preprint (which was not certified by peer review) is the author/funder, who has granted bioRxiv a license to display the preprint in perpetuity. It is made available under a CC-BY 4.0 International license.

Supplementary Figure 1

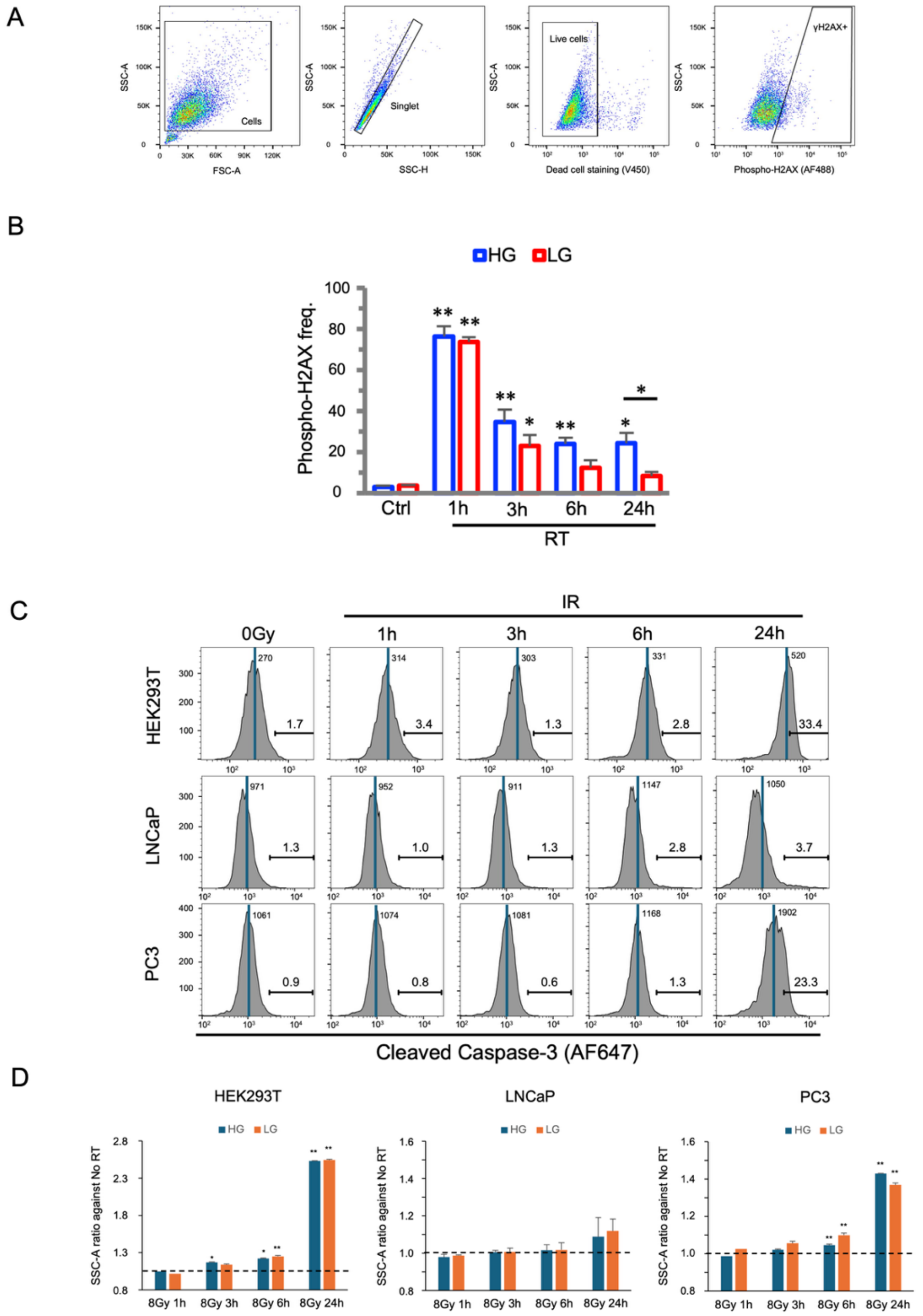

bioRxiv preprint doi: https://doi.org/10.64898/2025.12.03.690226; this version posted December 5, 2025. The copyright holder for this preprint (which was not certified by peer review) is the author/funder, who has granted bioRxiv a license to display the preprint in perpetuity. It is made available under a CC-BY 4.0 International license.

Supplementary Figure 2

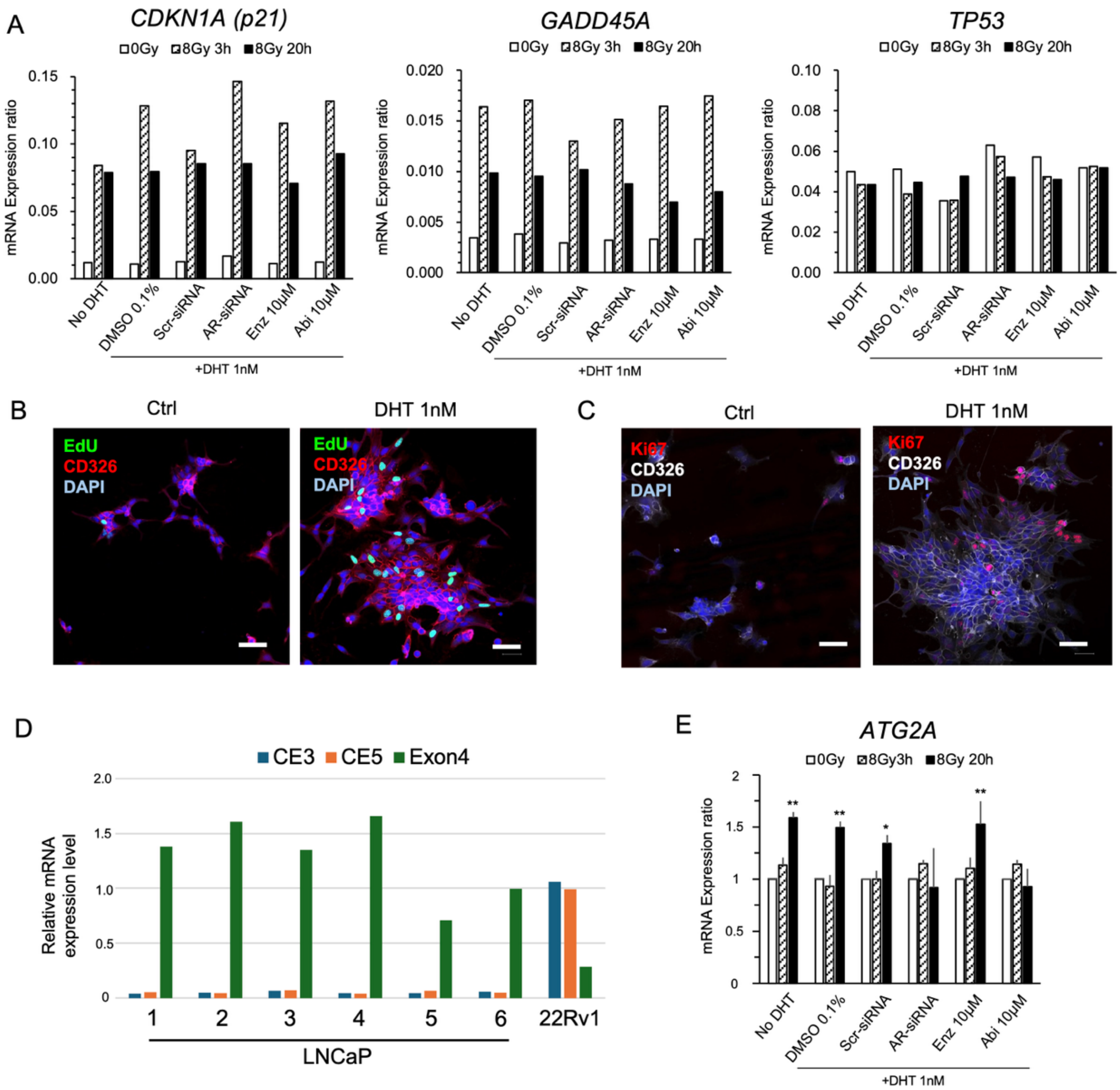

bioRxiv preprint doi: https://doi.org/10.64898/2025.12.03.690226; this version posted December 5, 2025. The copyright holder for this preprint (which was not certified by peer review) is the author/funder, who has granted bioRxiv a license to display the preprint in perpetuity. It is made available under a CC-BY 4.0 International license.

Supplementary Figure 3

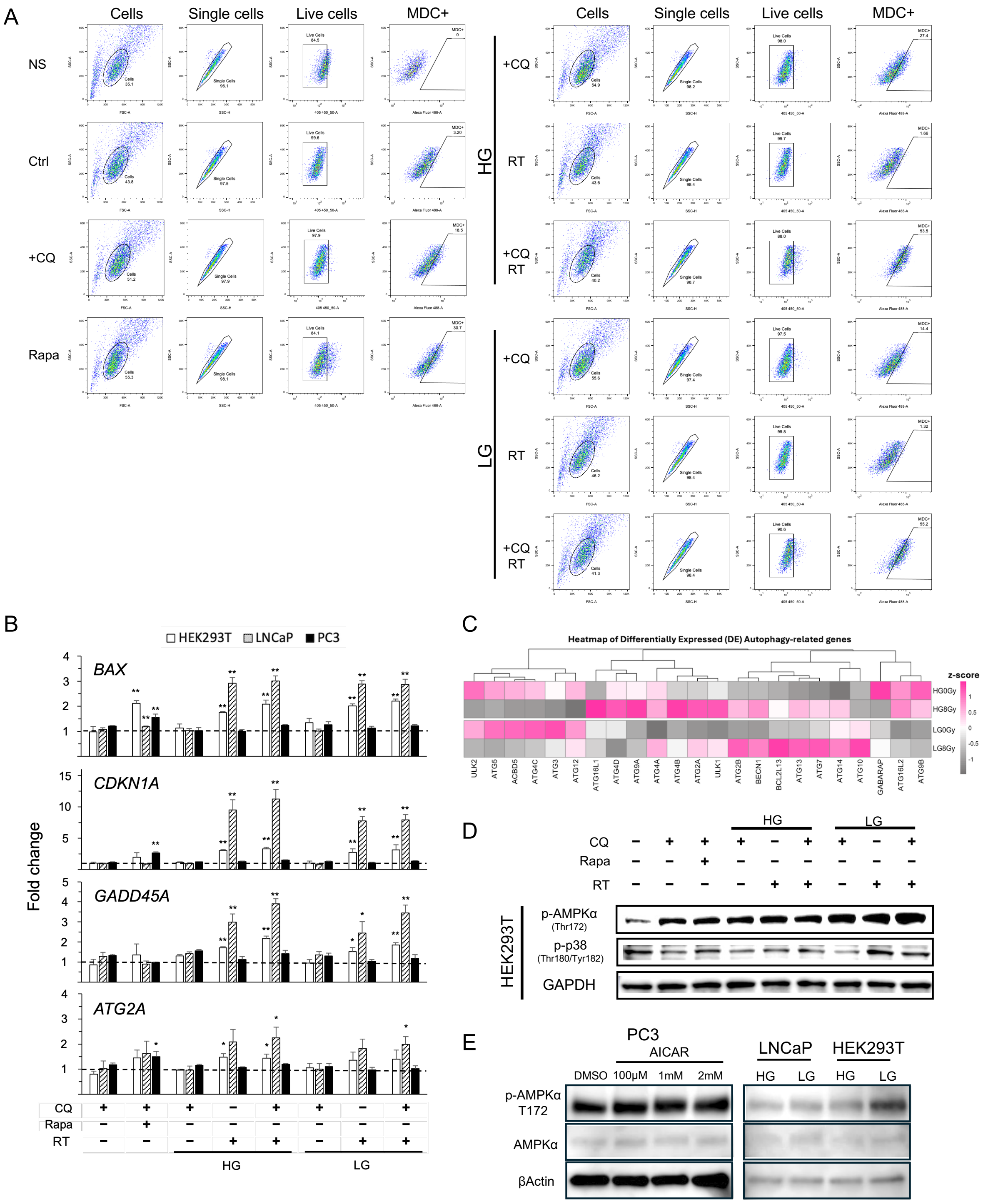

bioRxiv preprint doi: https://doi.org/10.64898/2025.12.03.690226; this version posted December 5, 2025. The copyright holder for this preprint (which was not certified by peer review) is the author/funder, who has granted bioRxiv a license to display the preprint in perpetuity. It is made available under a CC-BY 4.0 International license.

Supplementary Figure 4

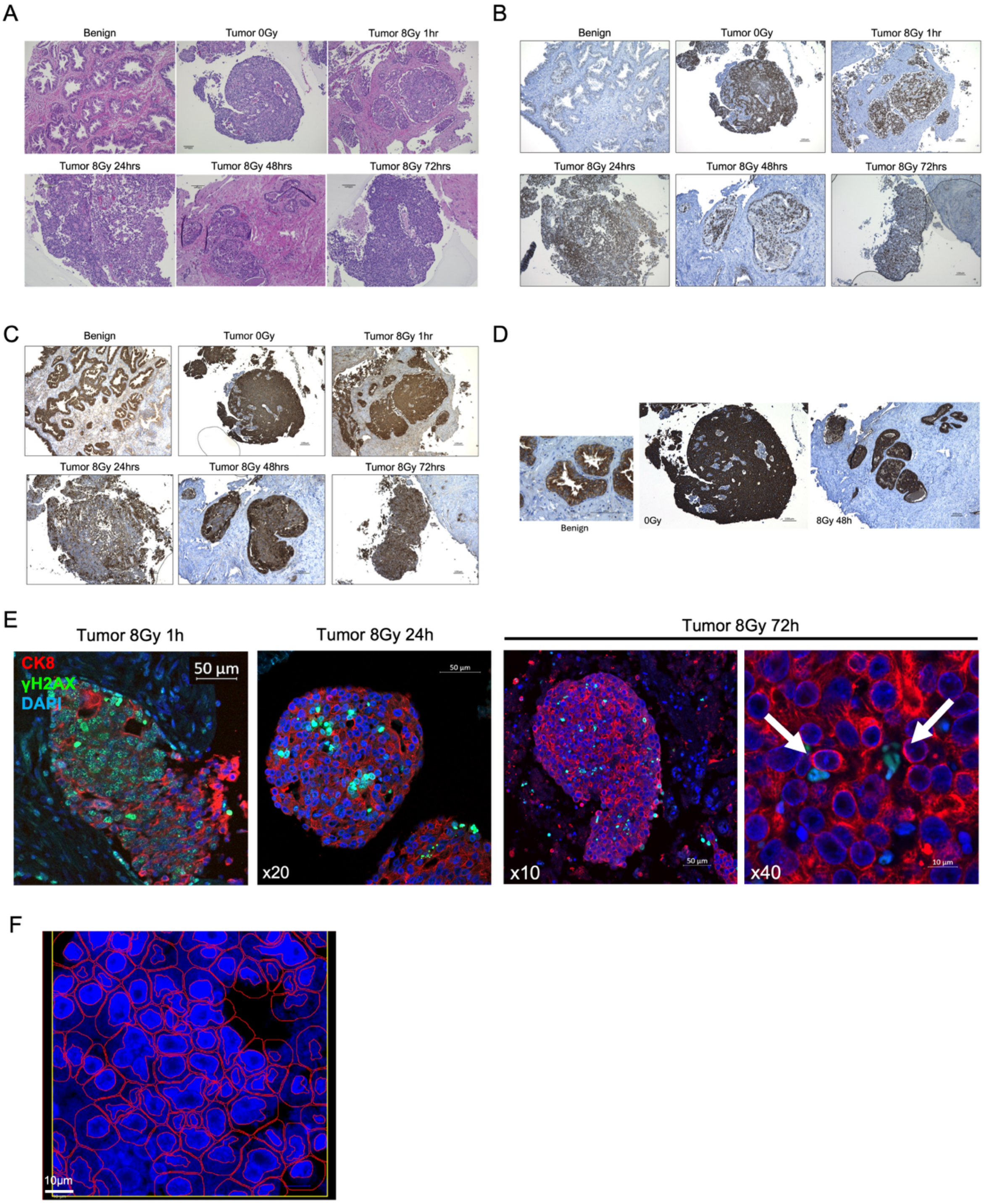

Supplement: Supplementary file 1 [file NIHPP2025.12.03.690226V1-supplement-1.pdf]
